# Will Healthcare Workers Accept a COVID-19 Vaccine When It Becomes Available? A Cross-Sectional Study in China

**DOI:** 10.3389/fpubh.2021.664905

**Published:** 2021-05-20

**Authors:** Yufang Sun, Xiaohong Chen, Min Cao, Tao Xiang, Jimei Zhang, Ping Wang, Hang Dai

**Affiliations:** Emergency Department, The Third People's Hospital of Chengdu, The Affiliated Hospital of Southwest Jiaotong University, The Second Affiliated Hospital Chengdu Clinical College of Chongqing Medical University, Chengdu, China

**Keywords:** COVID-19, vaccine, healthcare workers, vaccination, acceptance

## Abstract

**Objective:** The Coronavirus disease 2019 (COVID-19) vaccine is currently available. This timely survey was conducted to provide insight into on the willingness of healthcare workers (HCWs)to receive the vaccine and determine the influencing factors.

**Methods:** This was a cross-sectional online survey. An online questionnaire was provided to all participants and they were asked if they would accept a free vaccine. The questionnaire gathered general demographic information, and included the General Health Questionnaire (GHQ-12); Myers-Briggs Type Indicator questionnaire (MBTI); Depression, Anxiety, and Stress Scales (DASS-21); and the 12-item Short Form Health Survey (SF-12). The data were collected automatically and electronically. Univariate analysis was done between all the variables and our dependent variable. Multivariable logistic regression models were employed to examine and identify the associations between the acceptance of the COVID-19 vaccine with the associated variables.

**Results:** We collected 505 complete answers. The participants included 269 nurses (53.27%), 206 clinicians (40.79%), 15 administrative staff (2.97%), and 15 other staff (2.97%). Of these, 76.63% declared they would accept the vaccine. The major barriers were concerns about safety, effectiveness, and the rapid mutation in the virus. Moreover, four factors were significantly associated with the willingness to receive the vaccine: (a) “understanding of the vaccine” (odds ratio (OR):2.322; 95% confidence interval [CI]: 1.355 to 3.979); (b) “worried about experiencing COVID-19” (OR 1.987; 95% CI: 1.197–3.298); (c) “flu vaccination in 2020” (OR 4.730; 95% CI: 2.285 to 9.794); and (d) “living with elderly individuals” (OR 1.928; 95% CI: 1.074–3.462).

**Conclusions:** During the vaccination period, there was still hesitation in receiving the vaccine. The results will provide a rationale for the design of future vaccination campaigns and education efforts concerning the vaccine.

## Introduction

COVID-19 is caused by severe acute respiratory syndrome coronavirus 2 (SARS-CoV-2). It is currently the most urgent public emergency, which has attracted huge global attention. As of January 24, 2021, there have been a total of 99,152,664 confirmed cases, besides, 71,230,238 have recovered and 2,125,084 deaths have resulted all over the world. In China alone, there are official reports of 99,931 confirmed cases and 4,810 deaths as of January 24, 2021 (1). The pandemic has brought the danger of deaths from the epidemiologic contagion. Although drugs have been used to treat severe COVID-19 patients (2–5) and many policies have been in place to stop the spread of the virus, COVID-19 has continued to spread rapidly throughout the world. Therefore, vaccines for COVID-19 are considered an effective weapon to prevent the spread of the infection.

COVID-19 vaccines are finally becoming available, but uptake of any COVID-19 vaccine is an important challenge to address. A global survey found that 71.5% people would be very or somewhat likely to take a COVID-19 vaccine (6). One survey from July 2020 estimated that one-third or more of the United States (U.S). Population would decline COVID-19 vaccination (7). A cross-sectional study in Indonesia found that only 67.0% would like to be vaccinated if the effectiveness was 50% (8). A nationwide online survey in China from June 2020 revealed that 56.4% would be willing to receive the vaccine, with a definite yes intent of 28.7% (9). For medical institutions that are the main battlefield against the epidemic, protecting healthcare wokers (HCWs) against COVID-19 is crucial and some countries, including China have begun to carry out the mass vaccination campaign targeted at the highest risk groups including HCWs since December 2020 (10–12).

A cross-sectional study to assess the attitude of HCWs toward COVID-19 vaccination in U.S found that 36% of respondents were willing to take the vaccine when it became available while 56% were not sure or would wait to review more data (10). And a similar survey in Saudi Arabia revealed that 50.52% of HCWs were willing to have the COVID-19 vaccine (13). These investigations indicated that there is still some hesitation about vaccination when the vaccine become available, which could potentially blunt the potential of the vaccine in protecting long-term care residents.

Because HCWs are planned to be candidates for early vaccination and the role of HCWs becomes particularly important in advising patients and communities, and as well as through role modeling behavior. In this study, we assessed the acceptance of COVID-19 vaccine of HCWs in Third People's Hospital Of Chengdu in China and conducted a comparative analysis to examine what factors influence vaccination intentions. This study aimed to provide useful information to government and non-government organizations for taking the necessary steps toward a successful vaccination program.

## Materials and Methods

### Study Participants

A cross-sectional study was performed using the social media platform-based (WeChat) survey program, “Questionnaire Star,” between January 4 and 6, 2021. Participation was voluntary and the responses were anonymous. We included questionnaires from all HCWs from the Third People's Hospital of Chengdu (Sichuan, China). Those who agreed to participate in our study provided informed consent on the survey platform and later received a photo of a QR Code. They participated in the questionnaire by scanning this QR code. The exclusion criteria were as follows: (a) participants under 18 years old or over 59 years of age, (b) participants that were not HCWs in our hospital, and (c) participants that did not complete the assessment.

### Questionnaire and Data Collection

Before initiating the formal study, we first consulted psychologists working at our institution. The final questionnaire included an assessment of demographics (such as sex, age, education level, current position, marital status, children), the General Health Questionnaire (GHQ-12) (14–16), Myers-Briggs Type Indicator questionnaire (MBTI) (17–19), Depression, Anxiety, and Stress Scales (DASS-21) (20–23), and the 12-item Short Form Health Survey (SF-12) (24–27). This study was approved by the Ethics Committee of Third People's Hospital of Chengdu (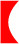
2021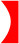-S-51), and all responders provided written informed consent before participating in this study. The survey lasted 3 days and ended on the day of vaccination. Incomplete questionnaires were eliminated electronically to ensure only full datasets were acquired. The data were collected through an online survey platform and the responses to the questionnaires were automatically encoded and organized by the “Questionnaire Star,” to avoid errors caused by manual entry. Finally, we exported the data to spreadsheets. The data were saved in both text format and numeric form.

### Description of the GHQ-12, MBTI, DASS-21, and SF-12

GHQ-12 is widely used in many studies to identify common psychiatric conditions (14, 15). According to the World Health Organization (WHO) guidelines, the GHQ-12 questionnaire is frequently used with the 0-0-1-1 scoring method where the first two and last two choices are scored as 0 and 1 points, respectively, leading to a total score ranging between 0 and 12 points. We used 3 points as the cut-off value, where 3 points or more suggested a mental health problem (16), the higher the score, the more significant the mental problem.

MBTI was developed to enable researchers to measure Jung's psychological types (17). It can measure Jung's three personality dimensions Extroversion/Introversion (E/I), Sensation/Intuition (S/N), and Thinking/Feeling (T/F), and also a dimension proposed by Myers, namely judging (J)/perceiving (P). MBTI is also frequently used to assess someone's personality (18, 19). In this study, we mainly discussed whether E/I or T/F would affect the vaccination intentions.

The DASS-21 is a popular measure of mental health (20, 21), and with its 21 items (7 items for each subscale) and three dimensions with similar psychometric properties is based on the tripartite model of depression, anxiety, and stress. Each 7-item subscale is rated on a 4-point Likert scale ranging from 0 (Did not apply to me at all) to 3 (Applied to me very much). Higher scores represent greater symptomology (22, 23).

The SF-12 has been used to investigate the quality of life (24, 25) and includes the Physical Component Summary (PCS) and Mental Component Summary (MCS) (26). The SF-12 physical (PCS-12) and mental (MCS-12) component summary scales were scored with reference to a formula (27). As it was particularly tedious to calculate the MCS and PCS singularly by individual items, we developed an EXCEL formula using Visual Basic for Applications(VBA)to process this data to avoid errors. The MCS and PCS scores ranged from 0 to 100, the higher the score, the better the quality of life.

### Statistical Analysis

All statistical analyses were performed using the SPSS 23.0 for Windows software package and the statistical significance level was set at a *p* ≤ 0.05. Descriptive statistics for the demographics and general health state of the medical staff were reported as the mean, standard deviation (SD), number (n), and percentage. Univariate analysis were done between all the variables and our dependent variable. Multivariable logistic regression models were employed to examine and identify the factors associated with the acceptance of COVID-19 vaccine.

## Results

### Demographic Characteristics

In this study, we collected a total of 505 responses, including 114 males (22.57%) and 391 females (77.43%).The participants included 206 clinicians (40.79%), 269 nurses (53.27%), 15 administrative staff (2.97%), and 15 other staff (2.97%). Of these, 97.42% had an educational level of bachelor's degree and above, 59.21% were married, 51.68% had at least one child, and 54.65% were living with an elderly individual. Of the participants, 61.19% (309) were extroverted. The demographic data are shown in Table 1.

**Table 1:** Univariate analysis showing factors associated with acceptance of a COVID-19 vaccine (*n* = 505).

**Variables**	***n***	**Group1**	**Group2**	***χ2/t***	***P*-value**
	**(505)**	**(*n* = 387)**	**(*n* = 118)**		
	**Frequency (%)**	**Frequency (%)**	**Frequency (%)**		
**Sex**					
Male	114(22.57)	93 (81.58)	21 (18.42)	2.011	0.168
Female	391(77.43)	294 (75.19)	97 (24.81)		
Age *(Mean ± SD)*	505	32.35 ± 8.98	32.71 ± 7.90	−0.395	0.693
Weight (KG) *(Mean ± SD)*	505	58.11 ± 11.16	56.99 ± 8.32	1.006	0.315
Height (cm) *(Mean ± SD)*	505	162.75 ± 7.06	162.68 ± 5.48	0.108	0.914
**Occupation**					
Clinician	206(40.79)	158 (76.70)	48 (23.30)	5.269	0.261
Nurse	269(53.27)	202 (75.09)	67 (24.91)		
Administration	15(2.97)	14 (93.33)	1 (6.66)		
Others	15(2.97)	13 (86.67)	2 (13.33)		
**Education**					
Junior/senior school*(R)*	13(2.58)	11 (84.62)	2 (15.38)	4.131	0.248
Bachelor	412(81.58)	321 (77.91)	91 (22.10)		
Postgraduate	80(15.84)	55 (68.75)	25 (31.25)		
**Marital status**					
Married	299(59.21)	225 (75.25)	74 (24.75)	0.783	0.394
Single	206(40.79)	162 (78.64)	44 (21.36)		
Children
Yes	261(51.68)	201 (77.01)	60 (22.99)	0.043	0.916
No	244(48.32)	186 (76.23)	58 (23.77)		
**Living with elderly individuals**					
Yes	276(54.65)	222 (80.43)	54 (19.56)	4.911	0.034^*^
No	229(45.35)	165 (72.05)	64 (27.95)		
**Flu vaccination in 2020**					
Yes	124(24.55)	114 (91.93)	10 (8.06)	21.491	0.000^*^
No	381(75.45)	273 (71.65)	108 (28.35)		
**Worried about experiencing COVID-19**					
Yes	334(66.14)	268 (80.24)	66 (19.76)	7.162	0.010^*^
No	171(33.86)	119 (69.59)	52 (30.41)		
**Understanding of the vaccine**					
Yes	401(79.41)	321 (80.05)	80 (19.95)	12.691	0.001^*^
No	104(20.59)	66 (63.46)	38 (36.54)		
**Effect of COVID-19**					
Not at all	265(52.48)	213 (80.38)	52 (19.62)	14.805	0.002^*^
Mild	160(31.68)	121 (75.63)	39 (24.38)		
Moderate	50(9.90)	28 (56.00)	22 (44.00)		
Severe	30(5.94)	25 (83.33)	5 (16.66)		
**GHQ-12**					
≥3	29(5.74)	20 (68.97)	9 (31.03)	1.010	0.365
<3	476(94.3)	367 (77.10)	109 (22.90)		
**MBTI (I/E)**					
Introvert	309(61.19)	237 (76.70)	72 (23.30)	0.002	1.000
Extrovert	196(38.81)	150 (76.53)	46 (23.47)		
**MBTI (T/F)**					
Thinking	266(52.67)	206 (77.44)	60 (22.56)	0.206	0.674
Feeling	239(47.33)	181 (75.73)	58 (24.26)		
Depression*(Mean ± SD)*	505	3.52 ± 3.99	3.45 ± 3.98	0.174	0.862
Anxiety*(Mean ± SD)*	505	3.73 ± 3.78	3.53 ± 3.57	0.510	0.611
Stress*(Mean ± SD)*	505	4.95 ± 4.45	4.81 ± 4.46	0.300	0.764
PCS *(Mean ± SD)*	505	52.34 ± 5.44	51.53 ± 6.81	1.329	0.184
MCS *(Mean ± SD)*	505	50.76 ± 8.57	50.33 ± 9.10	0.466	0.641

* *p < 0.05*.

### Acceptance of COVID-19 Vaccine and the Major Obstacles

**Figure 1:**
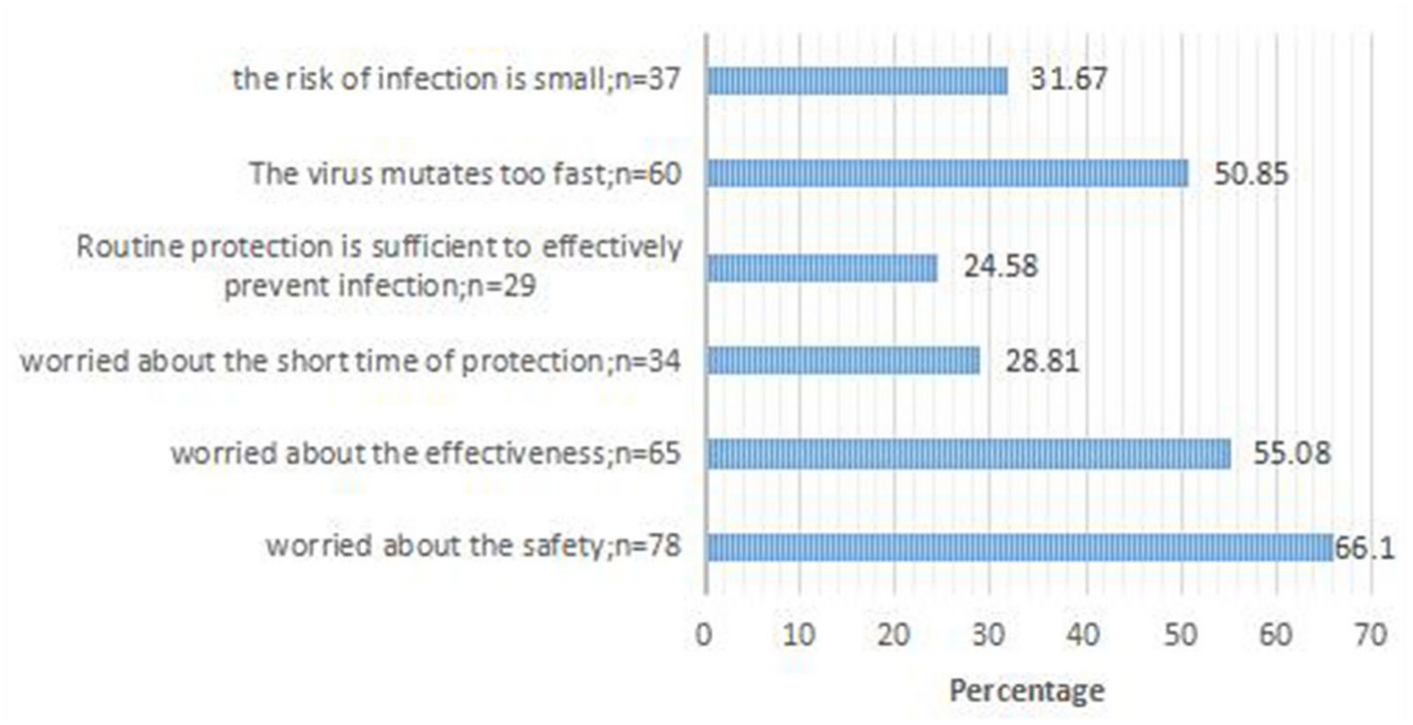
Reason for COVID-19 vaccine hesitancy; response for 118 participants who said they would refuse the COVID-19 vaccine.

Of the 505 respondents, 387 (76.63%) were willing to receive vaccination and 118 (23.37%) were not. We listed six possible factors. The top three reasons concerned safety, efficacy, and the rapid mutation of the virus (Figure 1).

### Variables Associated With the Acceptance of the COVID-19 Vaccine

For the univariate analysis (Table 1), we divided participants into two groups according to whether they were willing to be vaccinated, namely those who would accept a vaccine were put into group 1, while the remainder were placed in the second group. There were significant differences based on respondents living with elderly individuals, prior flu vaccination, understanding of the vaccine, worries of developing COVID-19, and the effects of COVID-19. Results of the group comparisons are displayed in Table 1. The results showed that individuals willing to receive a vaccine were more likely to be living with an elderly individual (χ^2^ = 4.911, *p* = 0.034), had a higher demand for flu vaccine (χ^2^ = 21.491, *p* = 0.000), were more worried about infection (χ^2^ = 7.162; *p* = 0.010), had a better understanding of the vaccine (χ^2^ = 12.691; *p* = 0.001), and believed COVID-19 had a greater impact on their lives (χ^2^ = 14.805; *p* = 0.002). However, there were no significant differences in terms of sex, age, occupation, educational level, marital status, personality, or physical and mental health status. Nonetheless, we found that men seemed to more likely accept a vaccine than women (81.58 vs. 75.19%), and the willingness to receive vaccination gradually decreased with the increase in educational level. According to the survey, we also found that clinicians and nurses seemed more hesitant to receive a vaccine compared with administrative and other staff members.

The Multivariable logistic regression regarding the factors that are associated with the willingness to be vaccinated is presented in Table 2.We found that four factors were significantly associated with the willingness to receive the vaccine: (a) “understanding of the vaccine”; (b) “worried about experiencing COVID-19”; (c) “flu vaccination in 2020” and (d) “living with elderly individuals.” Those who knew more about the vaccine properties were twice as likely to accept a COVID-19 vaccine, OR: 2.322; 95%CI: 1.355, 3.979, *p* = 0.002. In addition, those with high perceived risk to be infected had almost twice the odds of vaccine psychology compared to those with no perceived risk to be infected (OR: 1.987; 95%CI: 1.197, 3.298, *p* = 0.008). Those who get a Flu vaccine in 2020 were more likely to accept the vaccine compared to those who did not, with the OR: 4.730 (95%CI: 2.285,9.794, *p* = 0.000).Those living with elderly individuals had 1.928 times greater odds of accepting the vaccine compared to those who were not, OR: 1.928; 95%CI: 1.074, 3.462, *p* = 0.028. Those with a bachelor's degree were more likely to accept the vaccine compared to those with a Junior/senior school degree (OR: 2.353; 95%CI: (1.135, 4.880, *p* = 0.021). Those who thought the COVID-19 had severe effect on their lives were less likely to receive the vaccine than those who thought it had no effect (OR: 0.277; 95%CI:(0.084,0.913, *p* = 0.035).

**Table 2:** Multivariable logistic regression analyses showing factors associated with acceptance of a COVID-19 vaccine (*n* = 505).

**Variables**	**OR**	**95% CI**	***p*-value**
**Sex**			
Male*(R)*	1		
Female	0.761	0.398–1.457	0.410
Age *(Mean ± SD)*	0.977	0.940–1.016	0.241
Occupation			
Clinician*(R)*	1		
Nurse	0.472	0.070–3.192	0.442
Administrative staff	0.270	0.040–1.804	0.177
Others	0.858	0.052–14.267	0.915
**Education**			
Junior/senior school*(R)*	1		
Bachelor	3.699	0.511–26.750	0.195
Postgraduate	2.353	1.135–4.880	0.021*
**Marital status**			
Married *(R)*	1		
Single	1.442	0.637–3.265	0.380
**Children**			
Yes*(R)*	1		
No	0.506	0.207–1.233	0.134
**Living with elderly individuals**			
No*(R)*	1		
Yes	1.928	1.074–3.462	0.028*
**Flu vaccination in 2020**			
No*(R)*	1		
Yes	4.730	2.285–9.794	0.000*
**Worried about experiencing COVID-19**			
No*(R)* Yes	1		
	1.987	1.197–3.298	0.008*
**Understanding of the vaccine**			
No*(R)*	1		
Yes	2.322	1.355–3.979	0.002*
**Effect of COVID-19**			
Not at all*(R)*	1		
Mild	0.834	0.276–2.523	0.748
Moderate	0.591	0.193–1.812	0.358
Severe	0.277	0.084–0.913	0.035*
**GHQ score**			
<3*(R)*	1		
≥3	1.712	0.640–4.581	0.284
**MBTI (I/E)**			
Extroversion*(R)*	1		
Introversion	1.159	0.706–1.902	0.560
**MBTI (T/F)**			
Thinking*(R)*	1		
Feeling	0.848	0.524–1.374	0.504
Depression	0.988	0.880–1.110	0.840
Anxiety	1.020	0.897–1.160	0.764
Stress	1.028	0.923–1.145	0.611
PCS	1.041	0.996–1.087	0.074
MCS	1.009	0.978–1.041	0.580

## Discussion

Since the COVID-19 vaccine gradually become reality, some studies were conducted to assess acceptance of a COVID-19 vaccine (6–9). But most surveys have focused on the general population. In fact, since January 2021, our government has given priority to carry out the mass vaccination campaign targeted at the highest risk groups including HCWs, which also have happened in other countries (10–12). There are some studies showing that HCWs can themselves be vaccine hesitant and their hesitancy levels can thus impact hesitancy and aversion to receiving the vaccine among the general public (28–30). Therefore, we selected HCWs for this study. To the best of our knowledge, this cross-sectional study conducted during the early phase of the COVID-19 vaccination program is the first study evaluating the acceptance of a COVID-19 vaccine among healthcare wokers in China. In this study, we reported the proportion of HCWs willing to be vaccinated for COVID-19, and identified factors associated with acceptance of the vaccine. Our findings can be used to guide future projections of vaccine uptake.

In our study, three quarters of HCWs were willing to be vaccinated. The vaccination acceptance rate was higher compared to the similar studies conducted in Saudi Arabia and U.S (10). But the remarkable thing is we conducted our research in Chengdu while the other studies conducted in the whole country. In this study, only a quarter of respondents refused the COVID-19 vaccine. The major obstacles to accepting vaccination were concerns about side effects, the efficacy of the vaccine, and the potential for mutation of the virus, which were similar to the concerns raised in previous studies (31). These concerns are not surprising given the rapidity of vaccine development and its protective efficacy is still uncertain. Hence, public health intervention programs that focus on increasing the perception of the benefits of vaccination are needed. It is more important to improve the effectiveness and safety of the vaccine during the manufacturing process. We also found that some individuals who were not willing to vaccinate expressed optimism about the epidemic. This may have been a result of the effective control of the COVD-19 outbreak in our country. During the outbreak of the epidemic, Karaoke Television (KTV), bars, movie venues, and other businesses were closed, crowds were prohibited, workers were encouraged to hold online meetings, all individuals were required to reduce visits to relatives and friends during the Spring Festival, and were required to wear a facemask outdoors. This study did not take into consideration the cost of vaccination as a variable in the statistical analysis, because vaccines were freely available in China (32). In addition, the number of injections was not included in the analysis in this study because vaccines both nationally and abroad currently require two injections (33). Nonetheless, there may be additional reasons for an individual's refusal to vaccinate, which warrant future investigation.

Our study indicated that having more comprehensive knowledge about the vaccine might have contributed to them being more willing to accept the vaccine compared to those who had less information about the vaccine's properties. This suggested that greater education efforts about the vaccine should be considered to increase public confidence in vaccination. Additionally, our analysis also found that those who perceived themselves to be at risk for COVID-19 infection were more likely to accept the vaccine. Shekhar et al. (10) also reported similar findings in their HCWs population survey for COVID-19 vaccine uptake. Further, our study revealed that living with elderly individuals were associated with stronger intention to be vaccinated against COVID-19 though other studies have not included this factor. It is well-known that elderly individuals are more vulnerable to infectious diseases because of the considerable decline in the number of T-cells, which play an important role in identifying and reacting continuously to growing pathogens such as viral infections (34) which may have led staff members who are living with elderly individuals to accept the vaccine more readily in order not to transmit the virus to their family members. In addition, it is important to highlight that who had received flu vaccination in 2020 was a significant predictor of a definite willingness to be vaccinated for COVID-19 vaccination, as also suggested by Ameerah et al. (13).This may be in part due to the fact that they had already benefited from the experience of being vaccinated, and were also more likely to be more knowledgeable about the vaccine than others, and thus had a better understanding of its safety and side effects. We also found that those with a bachelor's degree were more likely to accept the vaccine compared to those with a Junior/senior school degree and those who thought the COVID-19 had severe effect on their lives were less likely to receive the vaccine than those who thought it had no effect, but it should be noted that there is a big difference in numbers between the two groups which could generate statistical error.

While prior work has explored the demographic and social underpinnings of decisions to receive a COVID-19 (6–8, 10, 13), little is known about how the physical and mental health state of people are associated with this choice. In fact, there have been some studies on the psychology of flu vaccination and those studies found that a sense of fear‵conspiratorial thinking were associated with vaccination (35, 36).In this study, we included in the questionnaire international scales to value general health state and personality of HCWs and we found that personality and physical and mental health status did not differ significantly between those willing to be vaccinated and those who were not. This indicated that the attitude toward vaccines is rational. If there is sufficient evidence provided to prove the effectiveness and safety of the vaccine, it is believed that the number of people willing to be vaccinated will increase significantly. This approach will help the government to successfully implement the prevention and control measures of COVID-19 and lay the foundation for the establishment of herd immunity.

A major strength of our study is that this study is the first study evaluating the acceptance of a COVID-19 vaccine among HCWs in China. In addition, we evaluated general health state and personality of responders to investigate if their physical and mental health state and personality would influence their hesitance to vaccination. These results indicated that the attitude toward vaccines is rational.

This study has a few limitations. First, our study employed an electronic questionnaire to collect data instead of a face-to-face questionnaire and is on a voluntary basis, resulting in sampling bias and uncontrolled conditions during the completion of the questionnaire. Moreover, the sample size was relatively small, thus the results should be considered preliminary and descriptive. Another limitation is that this study was conducted at a specific timepoint during the pandemic, and the results will likely change given the control of the spread of the virus and the development of a vaccine. Thus, further follow-up studies using qualitative and quantitative methods are necessary. Future studies will investigate whether and how propaganda and education can help HCWs to reduce their concerns about the vaccine. Furthermore, future studies should follow-up with the antibody test results of the HCWs who received the vaccine, and the protective effect of the vaccine.

## Conclusions

During the vaccination period, there was still hesitation in receiving the vaccine and specific concerns regarding COVID-19 vaccine are prevalent. In addition, willingness to be vaccinated was significantly associated with a better understanding of the vaccine's properties, perceived risk of COVID-19, prior flu vaccination in 2020, and living with elderly individuals. This study will help the government to better understand the social issues surrounding willingness for vaccination, and will improve publicity and education programs concerning the vaccine. In addition, the findings can provide a rationale for the design of future vaccination campaigns.

## Data Availability Statement

The original contributions presented in the study are included in the article/supplementary material, further inquiries can be directed to the corresponding author/s.

## Ethics Statement

This study was approved by the Ethics Committee of Third People's Hospital of Chengdu, and all responders provided written informed consent before participating in this study.

## Author Contributions

YS, TX, XC, and MC conceived and designed the questionnaire. TX, XC, PW, and JZ recruited participants. YS, TX, and HD analyzed the data. YS wrote and revised the paper. All the authors have approved the manuscript and agreed with submission to your esteemed journal.

## Conflict of Interest

The authors declare that the research was conducted in the absence of any commercial or financial relationships that could be construed as a potential conflict of interest.
